# Disease-associated gut microbiome and metabolome changes in rats with chronic hypoxia-induced pulmonary hypertension

**DOI:** 10.3389/fcell.2024.1022181

**Published:** 2024-07-12

**Authors:** Weitao Cao, Luyao Wang, Qiudi Mo, Fang Peng, Wei Hong, Yumin Zhou, Ruiting Sun, Haiqing Li, Chunxiao Liang, Dongxing Zhao, Mengning Zheng, Bing Li, Gongyong Peng

**Affiliations:** ^1^ State Key Laboratory of Respiratory Disease, Guangzhou Institute of Respiratory Health, The First Affiliated Hospital of Guangzhou Medical University, Guangzhou, China; ^2^ Department of Respiratory, The Second Affiliated Hospital of Guangzhou Medical University, Guangzhou, China; ^3^ Department of Critical Care Medicine, The Third Affiliated Hospital of Guangzhou Medical University, Guangzhou, China; ^4^ GMU-GIBH Joint School of Life Sciences, Guangzhou Medical University, Guangzhou, Guangdong, China; ^5^ Department of Thoracic Medicine, Shenzhen Second People’s Hospital, The First Affiliated Hospital of Shenzhen University, Guangzhou, China; ^6^ Department of Respiratory and Critical Care Medicine, Guizhou Provincial People’s Hospital, Guiyang, Guizhou, China

**Keywords:** chronic hypoxia, pH, gut microbiome, SCFAs, gut metabolome

## Abstract

**Background:**

Pulmonary hypertension (PH) is a progressive disease affecting the lung vasculature that is characterized by sustained vasoconstriction and leads to vascular remodeling. The lung microbiome contributes to PH progression, but the function of the gut microbiome and the correlation between the gut microbiome and metabolome remain unclear. We have analyzed whether chronic hypoxia-induced PH alters the rat fecal microbiota.

**Purpose:**

We explored hypoxia-induced pulmonary hypertension model rats to find out the characteristic changes of intestinal microorganisms and metabolites of hypoxia-induced pulmonary hypertension, and provide a theoretical basis for clinical treatment.

**Methods:**

In the current study, a chronic hypoxia-induced PH rat model was used to investigate the role of the gut microbiome and metabolome as a potential mechanism contributing to the occurrence and development of PH. 16S ribosomal ribonucleic acid (16S rRNA), short-chain fatty acid (SCFA) measurements, mass spectrometry (MS) metabolomics analysis and metatranscriptome were performed to analyze stool samples. The datasets were analyzed individually and integrated for combined analysis using bioinformatics approaches.

**Results:**

Our results suggest that the gut microbiome and metabolome of chronic hypoxia-induced PH rats are distinct from those of normoxic rats and may thus aid in the search for new therapeutic or diagnostic paradigms for PH.

**Conclusion:**

The gut microbiome and metabolome are altered as a result of chronic hypoxia-induced PH. This imbalanced bacterial ecosystem might play a pathophysiological role in PH by altering homeostasis.

## Introduction

Pulmonary hypertension (PH) is a pathophysiological disorder that involve multiple clinical factors and can be associated with a variety of cardiovascular and respiratory diseases. The mechanisms of PH are complicated and not well understood. Limited progress has been made in preventing or arresting the progression of PH despite extensive efforts (Humbert et al., 2022). Thus, it is imperative to consider innovative concepts for the discovery of new targets for a successful PH regimen.

There are five types in clinical classification of pulmonary hypertension: GROUP 1 Pulmonary arterial hypertension (PAH), GROUP 2 PH associated with left heart disease, GROUP 3 PH associated with lung diseases and/or hypoxia, GROUP 4 PH associated with pulmonary artery obstructions and GROUP 5 PH with unclear and/or multifactorial mechanisms (Simonneau et al., 2019). Although the causes of PH in the five groups are different, they can lead to pulmonary vascular pathological changes. Although the main lesion site of PH is the pulmonary vessels, it involves many other tissues and organs, such as the immune system (Parperis et al., 2023), heart (Cassady and Ramani, 2020), and kidney (Bolignano et al., 2013). The interaction of various organs causes PHs to have the characteristics of systemic diseases (Humbert et al., 2022). Recently, increasing evidence has shown that the intestinal tract and its microbial flora are closely related to lung diseases, especially chronic diseases. Changes in the intestinal microbial flora could affect the evolution of lung diseases, and in turn, the development of lung diseases also affects changes in the intestinal flora and its metabolites (Budden et al., 2019; Bowerman et al., 2020). Similarly, the “gut-lung axis” may play an important role in the pathogenesis of PH. Related papers have reported the microbiome of PAH and hypoxic PH. Daphne M Moutsoglou (Moutsoglou et al., 2023) has reported that the intestinal flora in the feces of PAH patients is dysbiotic, with an abundance of flora associated with inflammatory states and a low abundance of beneficial flora, and that the severity of pulmonary vascular disease is associated with a low diversity of intestinal microbiota. Observing the function and classification of the gut microbiome profile of PH patients showed that the gut microbes in PH patients differ from those in healthy individuals (Kim et al., 2020). It has been reported that there was no difference in the overall microbiome (α or β diversity) between SU5146 and hypoxia-induced PAH rats compared with normoxic rats, however, the ratio of Firmicutes and Bacteroidetes was significantly increased in SU5146 and hypoxia-induced PAH rats (Callejo et al., 2018; Sanada et al., 2020). With the emerging role of the gut microbiome in health and disease, the gut-lung axis could offer attractive alternative pathways in the pathogenesis of PAH or PH (Ranchoux et al., 2017; Wedgwood et al., 2020), as the gut and its microbiome play an important role in pulmonary vascular function. Although PAH and hypoxic PH have different etiologies, they share some common pathological mechanisms, such as increased oxidative stress in the pulmonary arteries, activation of HIF1a, and enhanced glycolysis in PASMCs. Hypoxia, as an important cause of pulmonary vascular remodeling and the development of PH, also has an important impact on intestinal microbes. We speculated that if the microbiome is changed in PAH, it might also be altered in hypoxic PH.

Therefore, we observed chronic hypoxia-induced pulmonary hypertension in rats. Thus, our objective in this study was to evaluate the hypothesis that chronic hypoxia-induced PH rats have a unique gut microbiome profile that produces microbial metabolites and molecules important in the pathogenesis of PH. Our data suggest that microbial imbalances may contribute to the development of chronic hypoxia-induced PH.

## Materials and methods

### Animal PH model

For the animal model, male Wistar rats that weighed from 250 to 350 g and were aged from 6 to 8 weeks were used in this study. The acquisition and care of rats are described in the Acknowledgments section. Twenty-nine rats were randomly divided into two groups: 15 in normoxia group and 14 in hypoxia-induced PH group. Rats were maintained in an environment with a controlled temperature (23°C) and relative humidity (60%) on a 12 h light and 12 h dark cycle and with free access to sterile food and water. For the hypoxia-induced PH model, male rats kept in normal chow were exposed to 10% O_2_ (hypoxia) or room air (normoxia) for 3 weeks as previously described (Lu et al., 2015). On day 21, sodium pentobarbital at a dose of 30 mg/kg was used to sacrifice the rats. The catheter was inserted into the isolated right external jugular vein, inserted into the right ventricle through the right atrium, and the right ventricular systolic pressure (RVSP) was recorded (Wu et al., 2020). The right ventricular hypertrophy index (RVHI) was measured by the ratio of the right ventricle/left ventricle + septum (Kim et al., 2010). Development of the PH model was evaluated by measuring RVHI and RVSP.

### Analysis of the gut microbiota

For analysis of the gut microbiota, fresh fecal samples were collected and processed from rats. Samples was further extracted using the OMEGA Soil DNA Kit (M5635-02) (Omega Bio-Tek, Norcross, GA, USA) by CTAB DNA extraction protocol (Arseneau et al., 2017), followed by sequencing on the Illumina HiSeq 2,500 platform. The primer sequences were as follows: 341F 5′-CCTAYGGGRBGCASCAG-3′ and 806R 5′-GGACTACNNGGGTATCTAAT-3′. Sequence assembly, quality control, and clustering were then performed.

### SCFA measurements

Quantification of SCFAs was performed as described previously (Garcia-Villalba et al., 2012). The column was an Agilent HP-INNOWAX capillary column (30 m*0.25 mm ID*0.25 μm) (Suzhou Bionovogene Co., Ltd); split injection, injection volume 1 μL, split ratio 10:1. The inlet temperature was 250°C; the ion source temperature was 230°C; the transfer line temperature was 250°C; and the quadrupole temperature was 150°C. The initial temperature of the temperature program was 90°C; then, it was heated to 120°C at 10°C/min and 150°C at 5°C/min; finally, it was heated to 250°C at 25°C/min and maintained for 2 min. The carrier gas was helium at a flow rate of 1.0 mL/min.

### Metabolomic profiling by liquid chromatography and mass spectrometry (LC‒MS)

Metabolomic analysis of all samples was performed using the Thermo Ultimate 3,000 system according to previous studies (Niu et al., 2019). Additionally, ESI-MSn analysis was conducted as previously described (Li et al., 2019; Niu et al., 2020). Briefly, chemical composition of rat fecal samples were evaluated the by untargeted LC-MS (liquid chromatography-mass spectrometry) form BioNovoGene (Suzhou, China). 100 mg of stool sample was added to 0.6 mL of 2-chlorophenylalanine in methanol and centrifuged at 12,000 rpm for 10 min at 4°C. We filtered out 300 μL of the supernatant using a 0.22 μM filter. Chromatography was performed using ACQUITY UPLC ^®^ HSS T3 (150 × 2.1 mm, 1.8 µm) (Waters, Milford, MA, USA) with the column temperature maintained at 40°C. The flow rate was set to 0.25 mL/min and the injection volume was set to 2 μL. Mass spectrometric detection of metabolites was used with an Orbitrap Exploris 120 (Thermo Fisher Scientific, USA). Electrospray ionization mass spectrometry (ESI-MSn) experiments were performed in positive and negative modes (voltages of 3.5 kV and −2.5 kV). The sheath, auxiliary gas, and capillary temperatures were set to 30 and 10 arbitrary units and 325°C. The analyzer’s scan mass range is m/z 81-1,000 with a mass resolution of 60,000.

### Metagenomic *de novo* sequencing

#### Extraction of total microbiome DNA

Mo Bio/QIAGEN’s DNeasy PowerWater Kit was used for extraction, and the extracted DNA was detected. Fluorescence spectrophotometer (Quantifluor-ST fluorometer, Promega, E6090; Quant-iT PicoGreen dsDNA Assay Kit, Invitrogen, P7589), measure the absorbance value of DNA at 260 nm and 280 nm respectively, detect the concentration of DNA, and use 1% agarose gel electrophoresis to detect the quality of DNA. Adjust the concentration of the DNA solution. Store the DNA working solution at 4°C and the storage solution at −20°C.

#### Library construction and sequencing process

Use the standard Illumina TruSeq DNA library preparation protocol (Illumina TruSeq DNA Sample Preparation Guide) to construct the required genomic on-machine library.

### Correlation analysis of the gut microbiome and host fecal metabolome

The correlation between the gut microbiota and the host fecal metabolome was analyzed. Analysis was performed as previously described (Duan et al., 2021). In brief, Pearson correlation analysis was performed using the coNet plug-in of the Cytoscape software to reveal the correlation between intestinal flora and host fecal metabolites, without setting the correlation coefficient and *p*-value threshold. Heat maps were used to display the correlation between intestinal flora and fecal metabolites.

### Statistical analysis

Data are shown as the mean ± standard error. Data were analyzed using Student’s t-test. Pearson’s correlation analysis was used to determine the correlation between the gut microbiota and the host fecal metabolome. Relative indices were analyzed using SPSS version 24.0 software. The data were graphically plotted using GraphPad Prism 7. Differences were considered statistically significant at *p* < 0.05.

## Results

### Changes in the gut microbiome in rats with chronic hypoxia-induced PH

**FIGURE 1:**
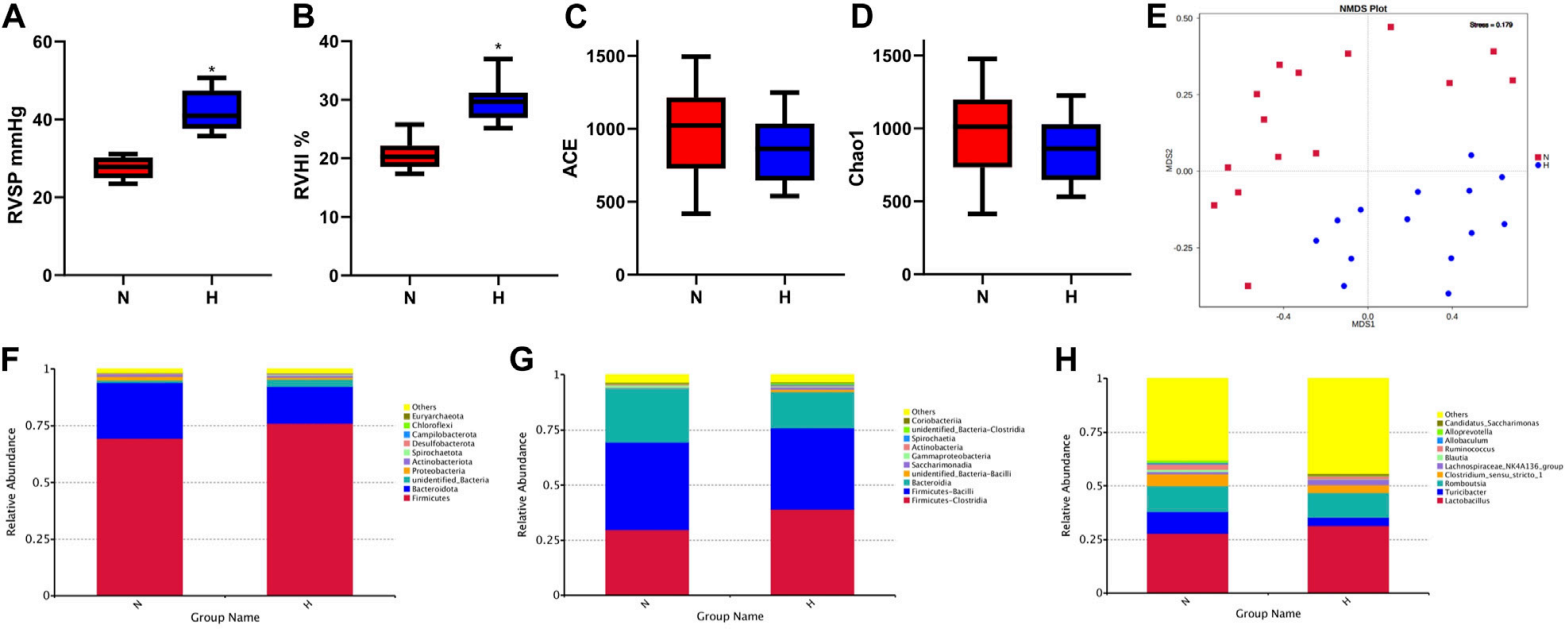
Intestinal microbial diversity and composition in the normoxia group and hypoxia-induced PH group. Differences in RVSP **(A)** and RVHI **(B)** between the two groups. Variation in diversity within the two groups by ACE **(C)** and Chao1 index **(D)**. NMDS plots based on **(E)**. Average relative abundances of dominant bacterial phyla **(F)**, classes **(G)**, and genera **(H)** in the intestine within the two groups. Normoxia group, *n* = 15; hypoxia-induced PH group, *n* = 14.

As a classic PH model of small animals such as rats, chronic hypoxia-induced PH is widely used to study the occurrence and development of PH (Dumas et al., 2012). PH induced by chronic hypoxia mainly caused the proliferation, hypertrophy and remodeling of rat pulmonary vascular smooth muscle, which led to thickened smooth muscle of the vascular media (Pak et al., 2007). Rats after 21 days of hypoxia, it can be seen that RVSP and RVHI in the hypoxia group were significantly higher than the normoxia group, indicating that the model was successful (Figures 1A, B). Chronic hypoxia is bound to be accompanied by changes in the gut microbiome of rats. We observed from the two indicators ACE and Chao1 in the alpha diversity index that compared with the normoxia group, the intestinal microbial composition diversity and richness of the hypoxia-induced PH group were lower (Figures 1C, D). In addition, based on the MNDS plot analysis, we identified different bacterial species in the two groups (Figure 1E). Although some bacterial genera of the two groups were similar in distance, they did not show obvious clusters. However, the abundance of intestinal flora changed in the two groups. At the phylum level, Firmicutes (*p* = 0.08), Spirochaetota (*p* = 0.07), Campilobacterota (*p* = 0.04), and Euryarchaeota (*p* = 0.21) were higher in abundance in the hypoxia-induced PH group than in the normoxia group (Statistically significant difference in Campilobacterota), but Bacteroidota (*p* = 0.02), Proteobacteria (*p* = 0.03), Actinobacteriota (*p* = 0.34), and Chloroflexi (*p* = 0.04) were lower in abundance than in the normoxia group (Statistically significant difference in Bacteroidota, Proteobacteria, Chloroflexi). At the class level, the abundances of Clostridia (*p* = 0.03), Saccharimonadia (*p* = 0.01), and Spirochaetia (*p* = 0.07) were increased in the hypoxia-induced PH group compared with those in the normoxia group (Statistically significant difference in Clostridia and Saccharimonadia), but the abundance of Bacteroidia (*p* = 0.02) was lower than in the normoxia group. At the genus level, the abundances of *Lactobacillus* (*p* = 0.49), Lachnospiraceae NK4A136 (*p* = 0.49), and Candidatus Saccharimonas (*p* = 0.01) were elevated in the hypoxia-induced PH group compared with those in the normoxia group (Statistically significant difference in Candidatus Saccharimonas), while the abundances of Blautia (*p* = 0.02), Ruminococcus (*p* = 0.04), Allobaculum (*p* = 0.12), Alloprevotella (*p* = 0.02), Turicibacter (*p* = 0.01)and *Clostridium* sensu stricto 1 (*p* = 0.45) were lower than in the normoxia group (Statistically significant difference in Blautia, Ruminococcus, Alloprevotella and Turicibacter) (Figures 1F–H).

**FIGURE 2:**
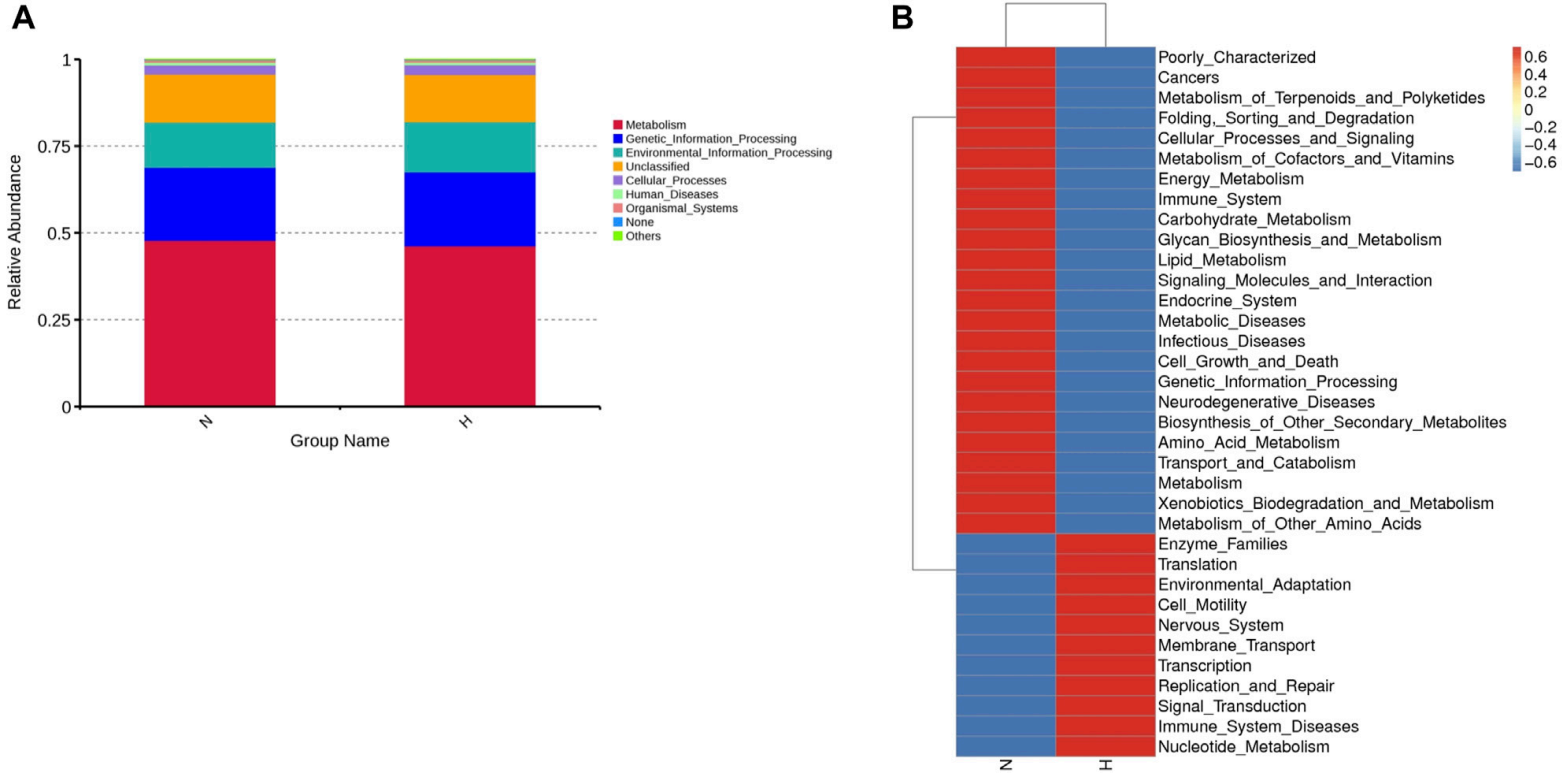
Differentially expressed genes involved in the pathogenicity of intestinal bacteria in the normoxia and hypoxia-induced PH groups. **(A)** Enriched genes involved in regulation. **(B)** Enriched genes listed in the heatmap. Normoxia group, n = 15; hypoxia-induced PH group, n = 14.

Using PICRUSt (version 1.1.4) to predict the function of intestinal microbes (Langille et al., 2013), it was found that the expression of some signaling pathways changed significantly, such as genetic information processing, metabolism processing and environmental processing. The functional genes that changed significantly in the hypoxia-induced PH and normoxic groups are shown in Figure 2.

### Changes in SCFAs in rats with chronic hypoxia-induced PH

**FIGURE 3:**
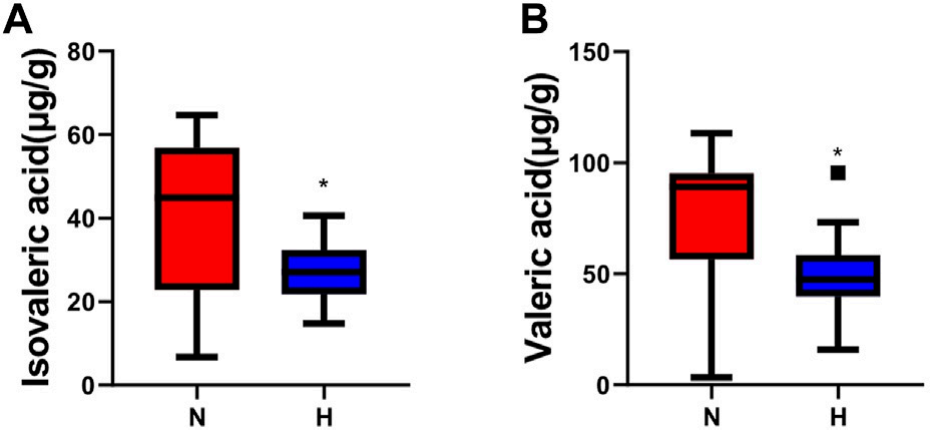
Changes in SCFAs in rats with chronic hypoxia-induced PH. The levels of isovaleric acid **(A)** and valeric acid **(B)** measured by GC‒MS in the normoxia group and hypoxia-induced PH group. The values are presented as the means ± SDs, *p < 0.05. Normoxia group, n = 15; hypoxia-induced PH group, n = 14.

Short-chain fatty acids (SCFAs) are important metabolites of intestinal microbes (Van Der Hee and Wells, 2021). They act as signaling molecules to affect a series of activities of the host. We observed that the primary SCFAs in rat feces were propionic, acetic, butyric and valeric acid and found that valeric acid and isovaleric acid levels in the hypoxia-induced PH group were lower than those in the normoxic group (normoxic group vs. hypoxia-induced PH group: valeric acid 74.17 ± 32.49 vs. 50.18 ± 19.49, *p* = 0.024; isovaleric acid 39.12 ± 17.66 vs. 27.26 ± 6.87, *p* = 0.026) (Figure 3).

### Changes in the fecal metabolome in rats with chronic hypoxia-induced PH

**FIGURE 4:**
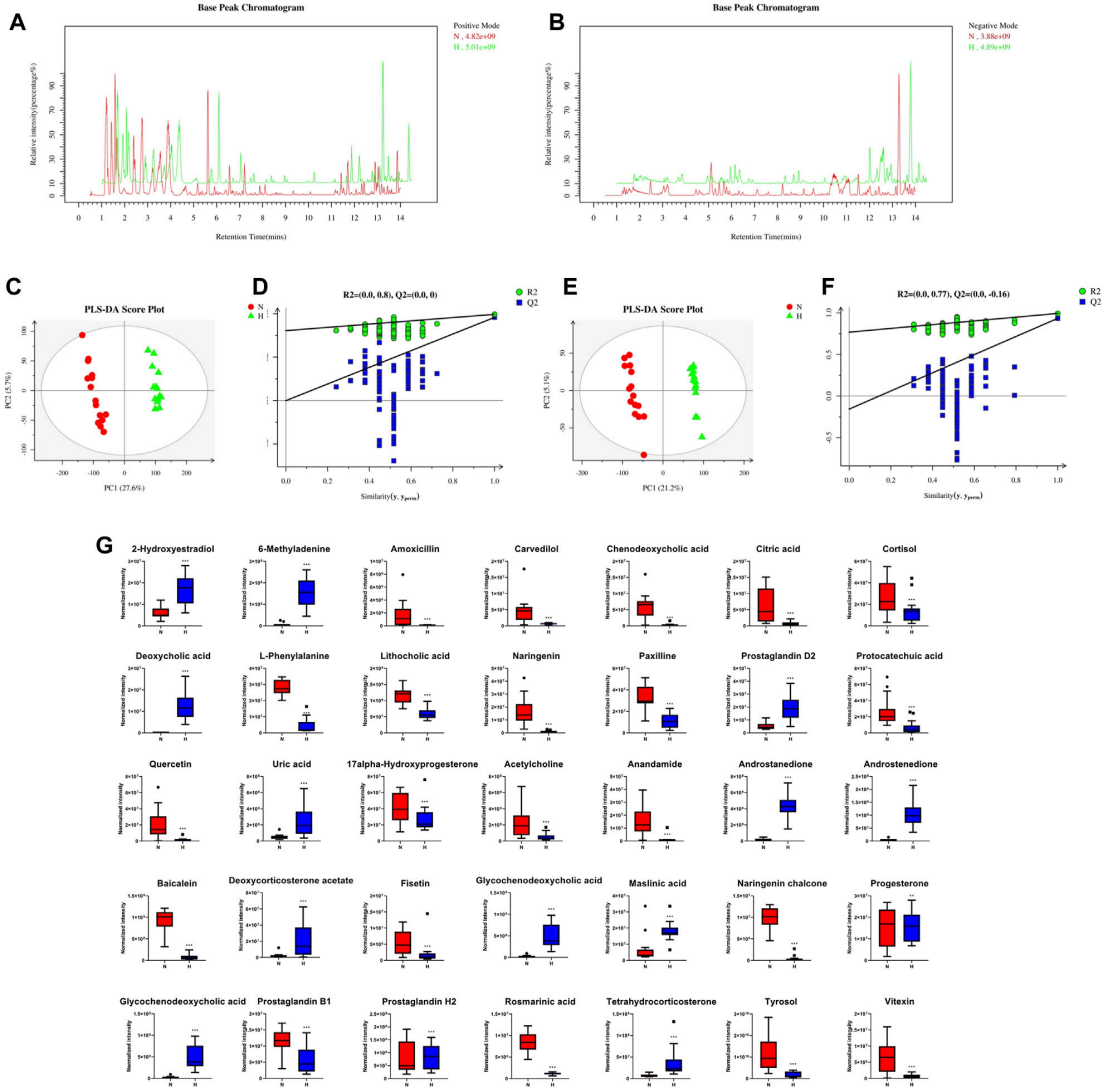
The effect of interference on the fecal metabolome. Typical LC–MS total ion current (TIC) chromatograms of nontarget metabonomics from the normoxia and hypoxia-induced PH groups in positive mode **(A)** and negative mode **(B)**. **(C)** PLS-DA score plot of positive ions. **(D)** Permutation testing of positive ions. **(E)** PLS-DA score plot of negative ions. **(F)** Permutation testing of negative ions. **(G)** Fecal metabolite disorder between the normoxia and hypoxia-induced PH groups. The data are presented as the mean ± SD, and significant differences between groups are indicated as ***p < 0.001, **p < 0.01 and *p < 0.05. Normoxia group, n = 15; hypoxia-induced PH group, n = 14.

**FIGURE 5:**
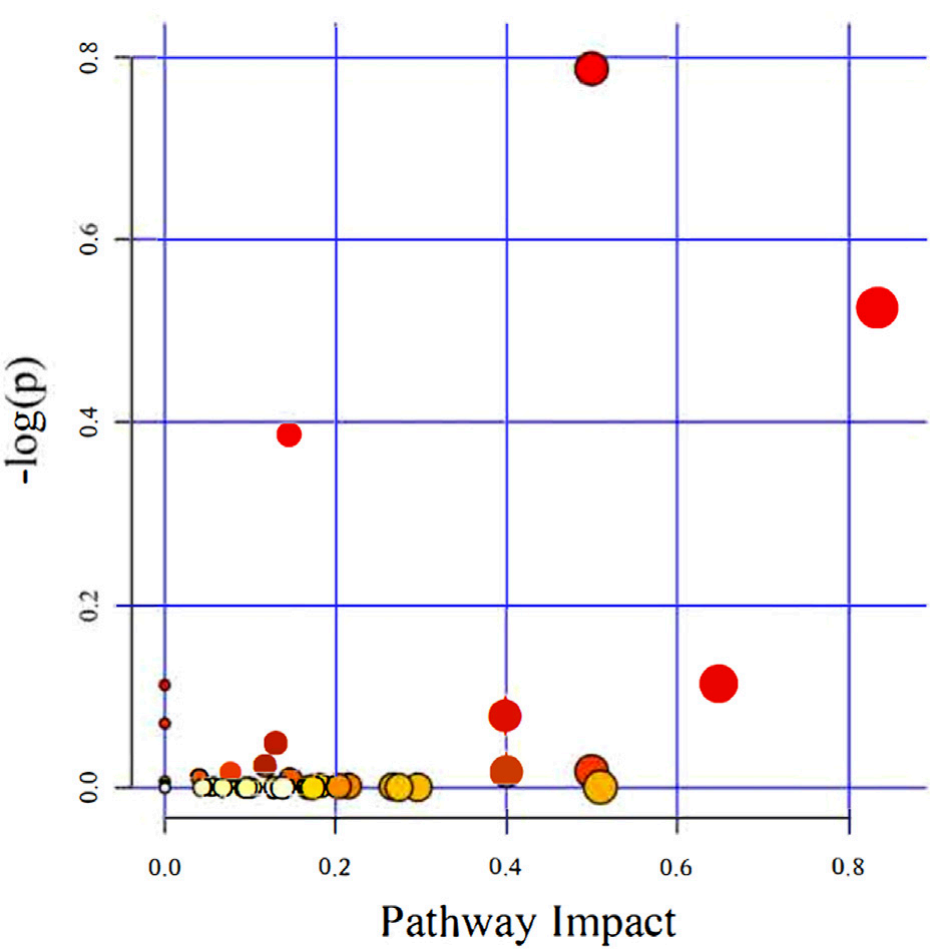
The KEGG pathways enriched and analyzed. Ingenuity pathway analysis. The size and color are based on the *p*-value and impact value, and a small *p*-value and large pathway impact value indicate that the pathway is greatly influenced.

Recently, studies have suggested that the occurrence and development of diseases are closely related to metabolites and that changes in intestinal flora may be related to changes in metabolites. Therefore, in addition to observing changes in intestinal flora, we also observed chronic hypoxia-induced PH rat changes in fecal metabolites. First, in positive and negative ion mode LC/MS total ion base peak chromatograms, it can be seen that the base peaks in the two groups are not at the same level, indicating that the metabolite abundances are different (Figures 4A, B). Next, to “simplify and reduce dimensionality” of high-dimensional and complex data on the basis of preserving the original information to the greatest extent and establish a reliable mathematical model to summarize the characteristics of the metabolic spectrum of the research object, we used partial least squares-discriminant analysis (PLS-DA) to screen for differential metabolites. As shown in Figures 4C–F, the contribution of each sample of PLS-DA to the observed component is equal and consistent. It was determined that the stability and repeatability of the analysis model were sufficiently good, so we used *p*-value ≤0.05 and VIP ≥1 as the screening criteria for differential metabolites. Our research found that compared with the normoxia group, many metabolites of rats in the hypoxia-induced PH group showed significant changes. Among the 35 metabolites, 14 metabolites were upregulated in the hypoxic group, and 21 metabolites were downregulated (Figure 4G). The intestinal balance in rats with hypoxia-induced pulmonary hypertension was disrupted, and the metabolites underwent significant changes. Furthermore, the method of KEGG annotation of the abovementioned significantly different metabolites enriched the signaling pathways (*p*-value and influence value), and the signaling pathways of biotin and phenylalanine metabolism had the most significant changes (Figure 5; Table 1). Our method is nontargeted metabolomics to support the overall change level in metabolomics predicted from related changes in microbiome composition.

**TABLE 1:** Result from pathway analysis.

	Total	Hits	Raw p	-log(p)	Holm adjust	FDR	Impact
Biotin metabolism	5	2	0.59131	0.52541	1	1	0.83334
Phenylalanine metabolism	9	2	0.89178	0.11454	1	1	0.64815
Steroid hormone biosynthesis	70	14	0.9991	0.000903	1	1	0.51027
Phenylalanine, tyrosine and tryptophan biosynthesis	4	2	0.45514	0.78714	1	1	0.5
Galactose metabolism	26	5	0.98194	0.018222	1	1	0.5
Methane metabolism	9	1	0.9823	0.017863	1	1	0.4
Tryptophan metabolism	41	11	0.92333	0.079764	1	1	0.39948
Glyoxylate and dicarboxylate metabolism	16	1	0.99925	0.000751	1	1	0.2963
Glycine, serine and threonine metabolism	32	4	0.99941	0.00059	1	1	0.27443
Tyrosine metabolism	42	7	0.99857	0.001433	1	1	0.267
Alanine, aspartate and glutamate metabolism	24	3	0.99789	0.002113	1	1	0.21519
Amino sugar and nucleotide sugar metabolism	37	6	0.99803	0.001968	1	1	0.20351
Arginine and proline metabolism	44	8	0.99745	0.002556	1	1	0.18142
Starch and sucrose metabolism	23	2	0.99955	0.000446	1	1	0.17244
Terpenoid backbone biosynthesis	15	1	0.99882	0.001182	1	1	0.17204
Pyrimidine metabolism	41	6	0.99947	0.000526	1	1	0.16844
Cysteine and methionine metabolism	28	5	0.99027	0.009773	1	1	0.14721
Histidine metabolism	15	5	0.67944	0.38649	1	1	0.14516
Aminoacyl-tRNA biosynthesis	67	5	1	4.57E-09	1	1	0.13793
beta-Alanine metabolism	19	4	0.9518	0.049396	1	1	0.12963
Steroid biosynthesis	35	4	0.99981	0.000194	1	1	0.12863
Arachidonic acid metabolism	36	8	0.97637	0.023914	1	1	0.11783
Porphyrin and chlorophyll metabolism	27	1	0.99999	5.02E-06	1	1	0.09987
Glutathione metabolism	26	3	0.99901	0.00099	1	1	0.09637
Drug metabolism - other enzymes	30	1	1	1.27E-06	1	1	0.09524
Drug metabolism - cytochrome P450	56	6	1	2.47E-06	1	1	0.09286
Vitamin B6 metabolism	9	1	0.9823	0.017863	1	1	0.07843
Primary bile acid biosynthesis	46	3	1	3.05E-07	1	1	0.06674
Pentose phosphate pathway	19	2	0.9977	0.002298	1	1	0.05556
Citrate cycle (TCA cycle)	20	1	0.99988	0.000122	1	1	0.05356
Glycerophospholipid metabolism	30	2	0.99998	2.35E-05	1	1	0.04444
Purine metabolism	68	6	1	2.40E-08	1	1	0.04305
Pantothenate and CoA biosynthesis	15	2	0.98869	0.011375	1	1	0.04082
Linoleic acid metabolism	5	1	0.89322	0.11292	1	1	0
Cyanoamino acid metabolism	6	1	0.93182	0.070613	1	1	0
Riboflavin metabolism	11	1	0.99281	0.007218	1	1	0
Nicotinate and nicotinamide metabolism	13	1	0.99708	0.002921	1	1	0
Pentose and glucuronate interconversions	14	1	0.99814	0.001858	1	1	0
Retinol metabolism	17	1	0.99952	0.000477	1	1	0
Fructose and mannose metabolism	19	1	0.99981	0.000193	1	1	0
Lysine degradation	20	1	0.99988	0.000122	1	1	0
Propanoate metabolism	20	1	0.99988	0.000122	1	1	0
Biosynthesis of unsaturated fatty acids	42	5	0.99992	8.00E-05	1	1	0
Sphingolipid metabolism	21	1	0.99992	7.76E-05	1	1	0
Glycolysis or Gluconeogenesis	26	1	0.99999	7.94E-06	1	1	0
Fatty acid biosynthesis	43	1	1	3.15E-09	1	1	0

“Total” is the total number of compounds in the pathway; Hits indicates the number of differential metabolites in target metabolic pathways; p is the original *p*-value calculated from the enrichment analysis; Holm p is the *p*-value adjusted by the Holm‒Bonferroni method; FDR p, is the *p*-value adjusted using the false discovery rate; Impact is the pathway impact value calculated from pathway topology analysis.

### Correlation between the gut microbiota and the fecal metabolome

**FIGURE 6:**
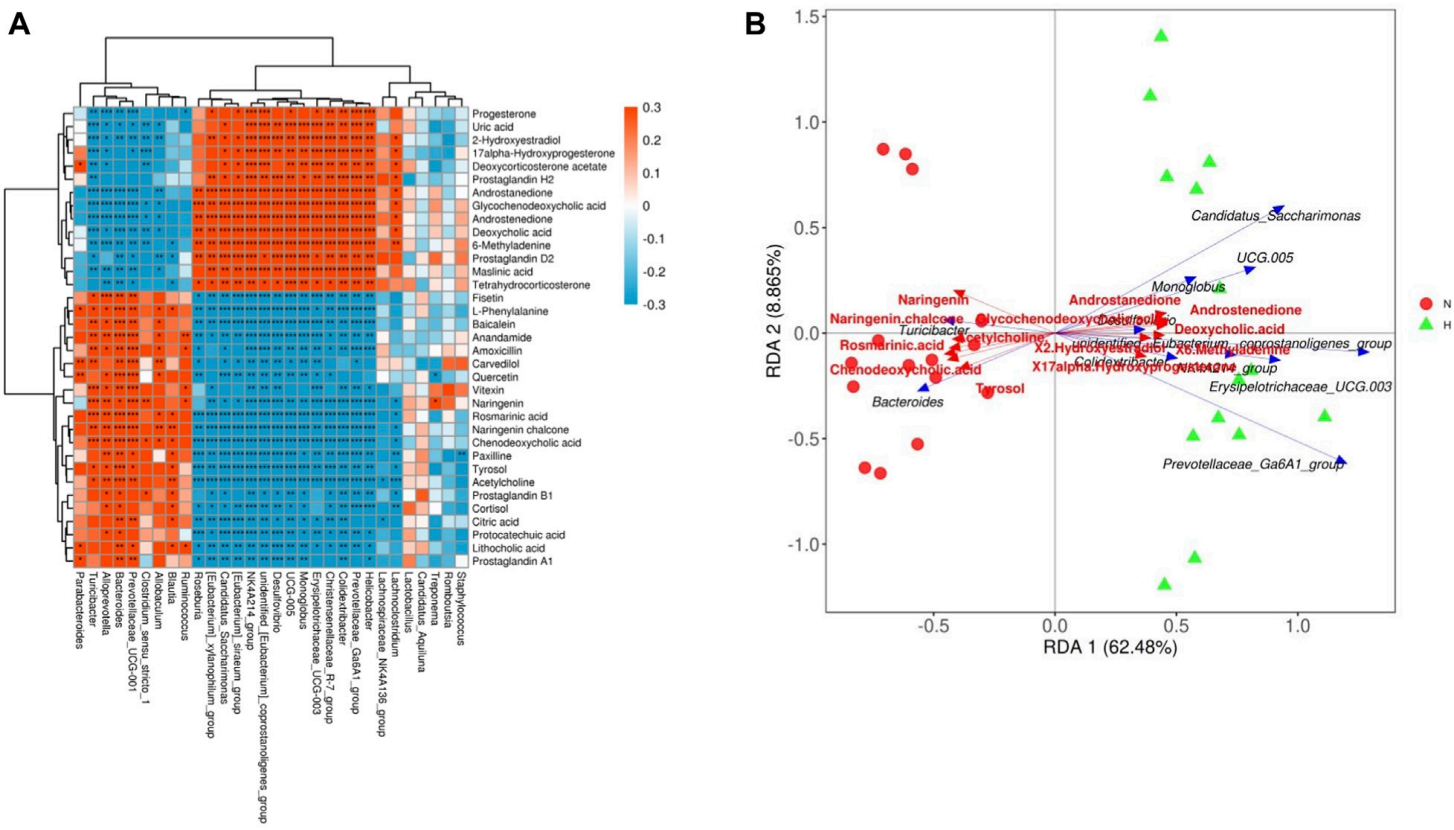
Relationship between the gut microbiome and host metabolome. **(A)** Heatmaps indicate positive (red) and negative (blue) correlations between the levels of host metabolites and the identified gut microbes at the genus level of normoxia rats compared with hypoxia-induced PH rats. The legend shows correlation values from −1 to 1 and assigns the appropriate color to them (red for positive correlations and blue for negative correlations). **(B)** Redundancy analysis between the levels of host metabolites and the identified gut microbiome at the genus level in normoxic rats compared with hypoxia-induced PH rats. There is an acute angle between the two variables, which represents a positive correlation, and there is a synergistic effect; there is an obtuse angle between the two variables, which represents a negative correlation, and there is an antagonistic effect. Normoxia group, n = 15; hypoxia-induced PH group, n = 14.

At present, an increasing number of studies report that gut microbes and their derived metabolites are the key mechanism for the communication and stimulation between gut microbes and the host, and it is an important link that affects the health of the host and the occurrence of diseases (Bowerman et al., 2020; Vernocchi et al., 2020; Gong et al., 2021). We explored the relationship between intestinal microbes and metabolites in rats with PH induced by hypoxia. After the results of the correlation heatmap and redundancy analysis, it can be seen that there are significant correlations between multiple gut microbiota at the genus level and 34 metabolic pathways. Figure 6 shows that among the 30 microbiota at the genus level, except for *Lactobacillus*, *Treponema*, Candidatus Aquiluna, Romboutsia, and *Staphylococcus*, the remaining gut microbiota had a strong correlation with 34 metabolites. These findings suggested relationships between gut microbiota changes and the host metabolome, which were promoted by hypoxia-induced PH development in rats.

### Changes in the fecal metatranscriptome in rats with chronic hypoxia-induced PH

**FIGURE 7:**
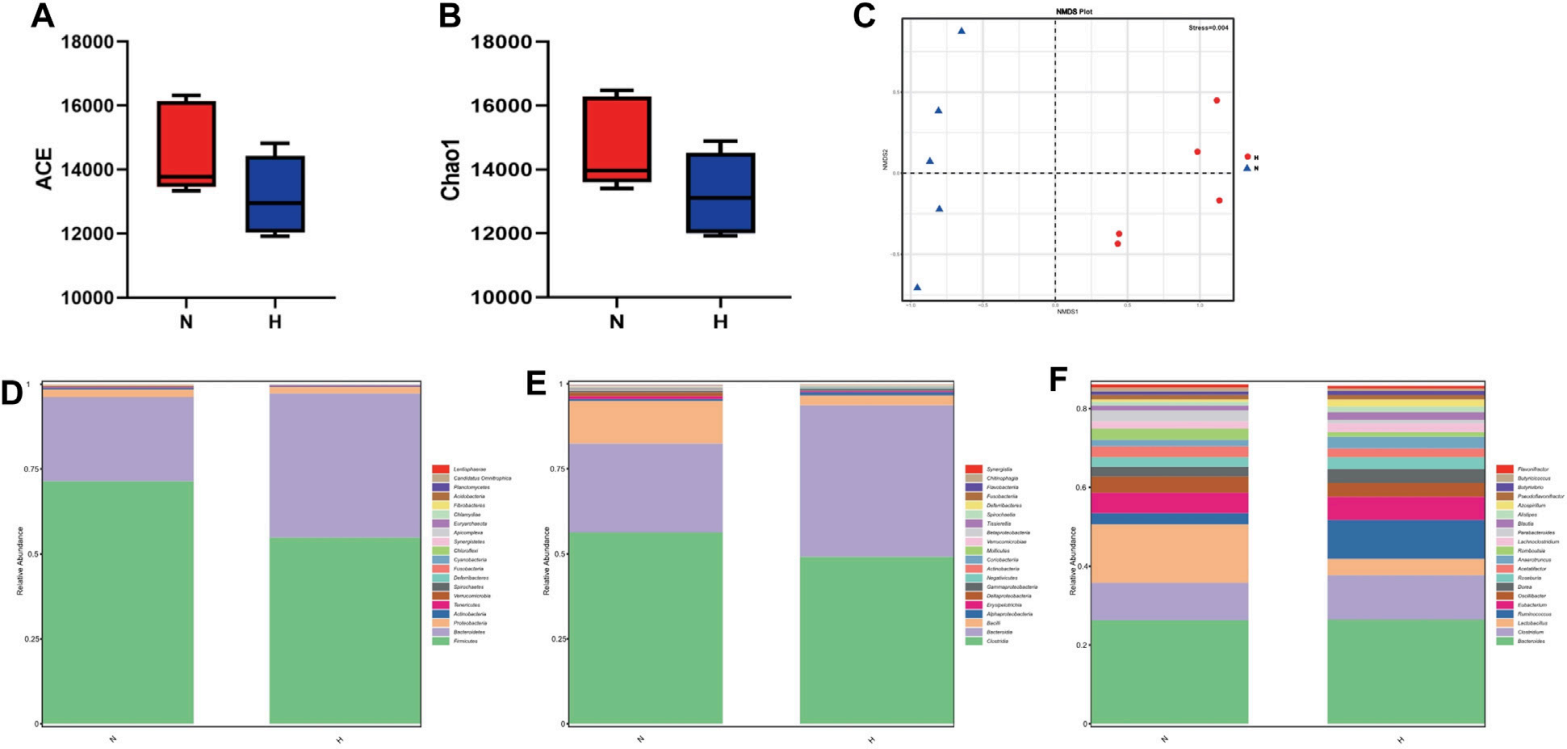
Metatranscriptome in intestinal microbial diversity and composition in the normoxia group and hypoxia-induced PH group. Variation in diversity within the two groups by ACE **(A,B)** Chao1 index. NMDS plots based on **(C)**. Average relative abundances of dominant bacterial phyla **(D)**, classes **(E)**, and genera **(F)** in the intestine within the two groups. Normoxia group, n = 5; hypoxia-induced PH group, n = 5.

**FIGURE 8:**
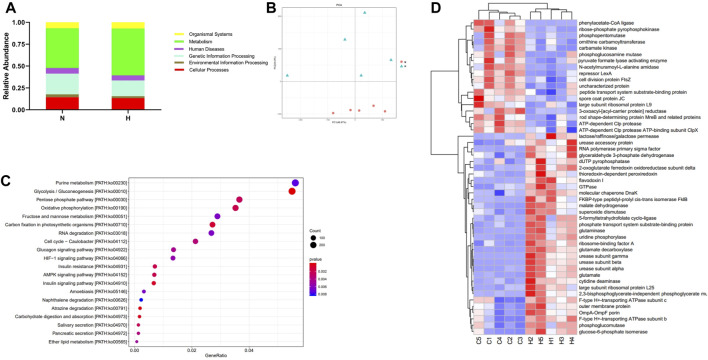
Metatranscriptomic analysis of intestinal microbial gene changes and functional changes in normoxia group and hypoxia-induced PH group. **(A)** Enriched genes involved in regulation. **(B)** PLS-DA analyzed differential metabolites between the two groups. **(C)** Differential functional unit clustering heat map. **(D)** KEGG metabolic pathway enrichment analysis. Normoxia group, n = 5; hypoxia-induced PH group, n = 5.

Metatranscriptomics mainly studies the expression levels of all transcripts (mRNA) of microorganisms in environmental samples and their transcriptional regulation rules under different environmental conditions at the population level, and studies the relationship between microorganisms and the natural environment. Metatranscriptome can study changes in complex microbial communities from the transcription level and can better explore potential new genes. First, we observed from two indicators, ACE and Chao1, that compared with the normoxic group, the intestinal microbial composition diversity and richness of the hypoxia-induced PH group were lower (Figures 7A, B). In addition, NMDS analysis showed that there was a significant difference in bacterial species between the two groups (stress = 0.004) (Figure 7C). Similarly, the abundance of intestinal flora in the two groups was different at the phylum and genus level. In addition, observing the function of intestinal microorganisms in the metatranscriptome, it was also found that the expression of some signaling pathways has changed significantly, such as genetic information processing, metabolism, and cells (Figure 8A). We used principal component analysis (PLS-DA) to analyze the differential metabolites between the two groups. It can be seen that there is a clear distinction between the two, indicating that there are significant differences (Figure 8B). For further functional difference analysis, we used the KO database to conduct KO metabolic pathway analysis on the respondent’s metatranscriptome. From the heat map results, we can see that many enzymes and proteins are significantly different between the two groups (Figure 8C). In addition, based on gene abundance, the KEGG metabolic pathway was also enriched from the metatranscriptome (Figure 8D).

## Discussion

It has been found that the intestinal microbes of patients with pulmonary arterial hypertension (type I PAH (Kim et al., 2020) and chronic thromboembolic pulmonary hypertension (Ikubo et al., 2022)) have significant changed, which are related to the pathogenesis of pulmonary arterial hypertension, but there are few studies on pulmonary hypertension caused by hypoxia. The study explored hypoxia-induced pulmonary hypertension model rats to find out the characteristic changes of intestinal microorganisms and metabolites of hypoxia-induced pulmonary hypertension, and provide a theoretical basis for clinical treatment.

With the continuous research being conducted on microorganisms, our understanding of their importance has become increasingly profound in the past 10 years. The remote gastrointestinal tract has the largest microbial reservoir in the human body, and its interaction with host cells affects the function of tissues and organs. At present, increasing evidence proves that the composition and function of the gastrointestinal microbiota are related to respiratory diseases. The mechanisms leading to the PH phenotype in MCT (monocrotaline) and hypoxia rat models were difference that MCT was related to endothelial toxicity and marked lung inflammation, whereas hypoxia was characterized by proliferative pulmonary vascular disease (Stenmark et al., 2009). Although significant changed in intestinal microbiota were found in MCT-induced PH rat models in our previous research (Hong et al., 2021), gut microbiome and metabolome changed in rats between MCT model and hypoxia model were difference. Therefore, it is necessary to elucidate the changes in gut microbiota and metabolites in chronic hypoxia-induced PH. In this study, 16S amplicon analysis technology was used to measure the flora in the rat intestine and mass spectrometry metabolomics to measure the changes in fecal metabolites. First, compared with the normoxia group, the hypoxia group’s gut microbiota alpha diversity and beta diversity were significantly reduced, which suggests that the hypoxia group’s intestinal diversity and richness are lower. Subsequently, we further observed the specific differences in the biological classification of the bacterial species in the two groups. The bacterial composition of the hypoxic group showed significant changes at the phylum, class, order, and genus levels. For example, in the hypoxic group, Turicibacter (Goodrich et al., 2016), Blautia (Liu et al., 2021), Alloprevotella (Cai et al., 2020) and other beneficial gut microbes related to inflammatory tumors changed significantly, which suggests that the health status of the hypoxic group was poor. These findings suggest that the imbalance of intestinal microbes may be related to pulmonary hypertension, which may be a risk factor for the development of pulmonary hypertension. Subsequently, we performed functional predictions of the gut microbiota and demonstrated host microbiome genes and related signaling pathway changes in hypoxia-induced PH rats, which suggested that hypoxia exposure increased the pathogenicity of the intestinal flora.

A stable gut microbiota plays a vital role in maintaining the homeostasis of barrier integrity, function, metabolism and immunity (Nicholson et al., 2012; Shi et al., 2017). However, alterations in the intestinal flora could lead to changes in the function of multiple organs in the host body (Bowerman et al., 2020). In this study, changes in intestinal microbes were accompanied by alterations in their metabolites. It has been found that valeric acid, an SCFA, can produce vasodilation and lower blood pressure (Onyszkiewicz et al., 2020), and we found that valeric acid and isovaleric acid were lower in the hypoxia-induced PH group. In the hypoxia-induced PH group, 35 significant changes in metabolites (14 upregulated, 21 downregulated) were closely related to the pathological changes in pulmonary vessels. For example, fisetin (Chen et al., 2019; Rodius et al., 2020), naringenin (Tang et al., 2017; Heidary Moghaddam et al., 2020), vitexin (Xue et al., 2020), baicalein (Woo et al., 2005), tyrosol (Boronat et al., 2019), quercetin (Ferenczyova et al., 2020) and other substances with protective effects on the cardiovascular system decreased significantly in the hypoxia-induced PH group. For prostaglandin H2 (Kato et al., 1990) and uric acid (Kimura et al., 2020), which promote vasoconstriction, inflammation and vascular sclerosis are significantly increased. Indeed, the microbiota not only impacted metabolites but also played a part in various signaling pathways. An increasing number of studies have shown that intestinal flora is related to various signaling pathways involved in the occurrence and development of PH (Chen et al., 2022). In this study, we enriched and identified 43 signaling pathways. and analyzed the change and functional influence of the intestinal flora on signaling pathways (Figure 5; Table 1). Among them, the biotin metabolism pathway was the most affected. We used metatranscriptomics to analyze rat feces, which also showed that the diversity and richness of intestinal microorganisms in the hypoxia-induced PH group decreased, and there were significant differences in metabolites and enriched KEGG metabolic pathways (Figure 8). Patients in each group of PH have the same pathophysiology, prognosis, and treatment response (Thenappan et al., 2018); however, there is heterogeneity among the groups. The potential mechanisms of PH may related to various causes such as chronic inflammation, immune disorders, and autophagy. It can be observed from the hypoxia-induced PH model in rats that the composition of the fecal microbiota changes. This unbalanced bacterial ecosystem may in turn play a role in the development of PH by changing immunity, inflammation, and metabolic homeostasis. It has been reported that there is also an enrichment of pro-inflammatory microbial metabolites in PAH patients (Moutsoglou et al., 2023). Therefore, this suggests that in patients with different types of PH, their fecal microbial groups and metabolite enrichments may be different. Fecal metabolites in rats induced by hypoxia may be different from those in PAH or other types of PH, but this change can also lead to increased production of metabolites such as pro-inflammatory substances, enrichment of signaling pathways such as ROS, biotin or HIF involved in the development of PH. These different changes can lead to the same result which may lead to participation in the development of PH through chronic inflammation, immunity and metabolism.

With the development of high-throughput sequencing technology and the continuous deepening of omics research, we can study the data integration of various omics techniques to comprehensively and systematically understand the interrelationship of multiple substances. This study integrates metabolomics and the microbiome, correlates metabolites with microbial abundance, species and genes, and analyzes the correlation between metabolites and microbial populations. Based on our study, exposure to hypoxia induced PH, and the gut microbiota may have a causal interaction with the host metabolome, which ultimately led to the correlation between the gut microbiota and the host metabolome. Such findings may further contribute to the development of novel biomarkers.

Our study has limitation. No human specimens were used in the study, and the clinical relevance is poor.

## Conclusion

We analyzed the stool of chronic hypoxia-induced PH rat through various methods such as: 16S ribosomal ribonucleic acid (16S rRNA), short-chain fatty acid (SCFA) determination, mass spectrometry (MS) metabolomics analysis and metatranscriptomics. The results showed that enriched signaling pathways of the intestinal microbiome and metabolome were significantly different from chronic hypoxia-induced PH rats to the normoxia group. Some signaling pathways are closely related to the onset of pulmonary hypertension caused by hypoxia. This imbalanced bacterial ecosystem might play a pathophysiological role in PH by altering homeostasis.

## Data Availability

The datasets presented in this study can be found in online repositories. The names of the repository/repositories and accession number(s) can be found below: NCBI BioProject accession number: PRJNA874536.
